# Robust Ferromagnetism in Hexagonal Honeycomb Transition Metal Nitride Monolayer

**DOI:** 10.3390/molecules29102322

**Published:** 2024-05-15

**Authors:** Xiaolin Ma, Zengqian Wang, Yuanfang Yue, Miao Gao, Fengjie Ma, Xun-Wang Yan

**Affiliations:** 1 College of Physics and Engineering, Qufu Normal University, Qufu 273165, China; 2 Department of Physics, School of Physical Science and Technology, Ningbo University, Ningbo 315211, China; 3 The Center for Advanced Quantum Studies and Department of Physics, Beijing Normal University, Beijing 100875, China; 4 Key Laboratory of Multiscale Spin Physics (Ministry of Education), Beijing Normal University, Beijing 100875, China

**Keywords:** ferromagnetism, two-dimensional materials, first-principles calculations, electronic structure, Curie temperature

## Abstract

Two-dimensional intrinsic magnetic materials with high Curie temperature are promising candidates for next-generation spintronic devices. In this work, we design two kinds of two-dimensional transition metal nitrides, VN_2_ and FeN_2_, both with a hexagonal honeycomb lattice. Based on the formation energy, and phonon spectra calculations as well as the molecular dynamics simulations, their structural stability is demonstrated. Then, we determine the ferromagnetic ground states of VN_2_ and FeN_2_ monolayers through the energy calculations, and the Curie temperatures of 222 K and 238 K are estimated by solving the Heisenberg model using the Monte Carlo simulation method. Hence, the VN_2_ and FeN_2_ monolayers are demonstrated to be new two-dimensional ferromagnetic materials with high temperature ferromagnetism or large-gap half-metallicity.

## 1. Introduction

Because of the novel mechanical, thermal, electronic, and magnetic properties associated with the reduced dimensionality, two-dimensional (2D) materials have attracted a lot of research interest in physics and materials. Since graphene was discovered in 2004 [1], various 2D materials have been fabricated in experiments, such as boron nitride [2], silicene [3], borophene [4], and transition metal dichalcogenides [5]. Recently, more and more attention has been paid to 2D transition metal nitrides, because they have good mechanical and electronic properties, include flexibility and suitable energy band gap [6,7,8,9,10,11].

Some MXenes [12] are typical 2D transition metal nitrides, which commonly appear as M_2_N, M_3_N_2_, and M_4_N_2_ (M is a transition metal atom) [13]. In the M_2_N (M = Ti, Cr, and Zr) monolayers, one layer of N atoms is sandwiched between two layers of M atoms. If the two elements of the M_2_N layer are exchanged, is the new structure of the MN_2_ layer stable? After the Surface modification with the OH moiety or O atom, the nonmagnetic Cr_2_N monolayer can become the ferromagnetic Cr_2_N(OH)_2_ or Cr_2_NO_2_ monolayer [14,15]. In them, the Cr layer is between the N and O layers. Inspired by the emergence of ferromagnetism in Cr_2_N(OH)_2_ and Cr_2_NO_2_, a question is raised. If one layer of metal atoms is intercalated between two layers of N atoms, namely the MN_2_ structure mentioned above, would the ferromagnetism emerge? Although the initial idea to build the 2D ferromagnetic monolayer is simple, there are two facts to motivate us to further explore the 2D ferromagnetism in the MN_2_ systems. In the previous research on the 2D transition metal nitrides, MoN_2_, ReN_2_, and TaN_2_ have been predicted and synthesized [10,16,17]. From this, we infer that the MN_2_ compounds are probably also stable when M is 3d transition metal atom. On the other hand, the M atoms in the MN_2_ structure form a triangle lattice, which is more preferred for ferromagnetic order than antiferromagnetic order because the antiferromagnetic interactions are usually frustrated in a triangle lattice.

The Mermin-Wagner theorem states there is no long-range 2D magnetic order at any finite temperature in an isotropic spin orientation case [18]. This theorem can lead to the misleading impression that it is difficult to prepare magnetic two-dimensional materials experimentally and then hinder the experimental development of the magnetic 2D materials. In fact, the long-range 2D magnetic order can exist in a 2D materials when the isotropic spin orientation is broken. Until 2017, the 2D ferromagnetism in CrI_3_ monolayer with the Curie temperature of 45 K was discovered [19], which is the first experimental realization of the magnetic long-range order in a 2D material. The milestone discovery has stimulated great research interest in the ferromagnetism in 2D materials. Subsequently, 2D Cr_2_Ge_2_Te_6_ [20] was synthesized, and the ferromagnetism with the Curie temperature of about 30 K was measured by scanning magneto-optic Kerr microscopy. In the 2D VSe_2_ layer synthesized by Bonilla et al. [21], the room-temperature ferromagnetism was observed. Recently, in atomically thin Cr_2_Te_3_, a conspicuous ferromagnetic transition from Stoner to Heisenberg-type is directly observed, indicating that the Heisenberg model is suitable to describe the magnetic interactions in 2D magnets with the dual nature of localized and itinerant ferromagnetism [22]. Anomalous Hall effect was confirmed by magneto-transport measurements in single-sheet 2D Fe_3_GaTe_2_ crystals with room-temperature ferromagnetism [23]. In the CoPS_3_, FePS_3_, and NiPS_3_ 2D layers with an antiferromagnetic zigzag order, the magneto-elastic response was investigated and spontaneous anisotropic magnetostriction was found [24]. Magnetic 2D materials offer an emerging platform for fundamental studies of magnetism in the 2D limit. In addition, the Curie temperature is a vital parameter of ferromagnetic 2D materials, and the low Curie temperature is the main obstacle limiting the application of ferromagnetic 2D materials in electronic devices. Up to now, ferromagnetic 2D materials with high Curie temperatures are scarce. Therefore, it is an urgent and necessary task to explore new two-dimensional ferromagnetic materials for both fundamental physics studies and application research on functional materials.

In this work, we propose two new ferromagnetic 2D transition metal nitrides, VN_2_ and FeN_2_ monolayers, and systematically investigate their dynamical, electronic, and magnetic properties. Based on the Heisenberg model and the Monte Carlo simulations, the Curie temperatures are estimated, and the high-temperature ferromagnetism in the 2D FeN_2_ and VN_2_ layers is demonstrated.

## 2. Results

### 2.1. Atomic Structure

**Figure 1:**
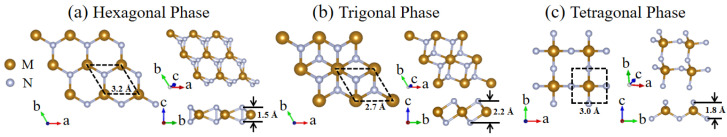
Top view, oblique view, and side view of the MN_2_ (M = V and Fe) structures in the hexagonal (**a**), trigonal (**b**), and tetragonal (**c**) phases.

We design three structures of MN_2_ (M = V and Fe) with the M layer intercalated between two N layers, shown in Figure 1a–c. The first structure is a hexagonal (H) phase with the symmetry of the P$\bar{6}$m2 (No. 187) space group, in which the M atom is coordinated by six N atoms and located at the center of a regular triangular prism of six nitrogen atoms. This is the hexagonal honeycomb geometry, similar to the well-known graphene, boron nitride, and MoS_2_ monolayers. The second structure is a trigonal (T) phase with the symmetry of the P$\bar{3}$m1 (No. 164) space group, in which the M atom is in an octahedral configuration bonded to six N atoms. The third structure is a tetragonal phase (Tetra) with the symmetry of the P$\bar{4}$m2 (No. 115) space group, in which the M atom is at the center of a tetrahedron formed by four N atoms. Table 1 presents the energies of the MN_2_ (M = V and Fe) monolayers in the hexagonal, trigonal, and tetragonal phases. As shown, the hexagonal phase has the lowest energy and is determined to be the most likely structural phase of the 2D MN_2_ (M = V and Fe) compounds. In the hexagonal MN_2_ monolayer, the N-N distances are 1.568 Å and 1.336 Å for VN_2_ and FeN_2_, respectively, which are close to the length of the N-N single bond, 1.46 Å. The formation of the N-N bond is the main reason why the hexagonal structure has a lower energy compared to the trigonal and tetragonal phases. So, the hexagonal VN_2_ and FeN_2_ monolayers are selected as our research objects, and the mechanical, electronic, and magnetic properties are investigated in the following sections.

### 2.2. Evidence for Structural Stability

As analyzed in the above paragraph, the hexagonal phase of FeN_2_ and VN_2_ layers is the low-energy structural phase, in which we note that one unit cell only contains three atoms, one Fe (or V atom) and two N atoms. To further inspect the structural stability of hexagonal FeN_2_ and VN_2_ monolayers, we compute the formation energies with the standard generalized gradient approximation in the Perdew- Burke-Ernzerhof form. The formation energy per atom is expressed as

(1) $$E_{form}=\left(E_{MN_{2}}-E_{M}-E_{N_{2}}\right)/3,$$

in which *E*_*MN*_2__ is the energy of one unit cell of the MN_2_ monolayer, *E_M_* is the energy of one metal atom in the bulk metal, *E*_*N*_2__ is the energy of a nitrogen molecule, and the number 3 is the total number of atoms in one unit cell. in which *E*_*MN*_2__, $E_{M}$, and *E*_*N*_2__ are the energies of the MN_2_ monolayer, bulk metal per atom, and nitrogen molecule, respectively. The formation energy averaged over the number of atoms is −0.113 eV/atom and 0.665 eV/atom for FeN_2_ and VN_2_, respectively. Because N_2_ molecules have low energy due to their N≡N triple bond, metal nitrides usually have positive formation energies. For example, the formation energies of the nitrides CuN_3_ [25], PtN_2_ [26] and g-C_3_N_4_ [27] that have already been synthesized experimentally are 0.42 eV/atom, 0.55 eV/atom, and 0.35 eV/atom, respectively. The formation energies of the 2D FeN_2_ and VN_2_ monolayers are comparable to the ones of these nitrides, which indicates that similar to the CuN_3_, PtN_2_, and g-C_3_N_4_, the FeN_2_ and VN_2_ monolayers can exist under ambient conditions.

**Figure 2:**
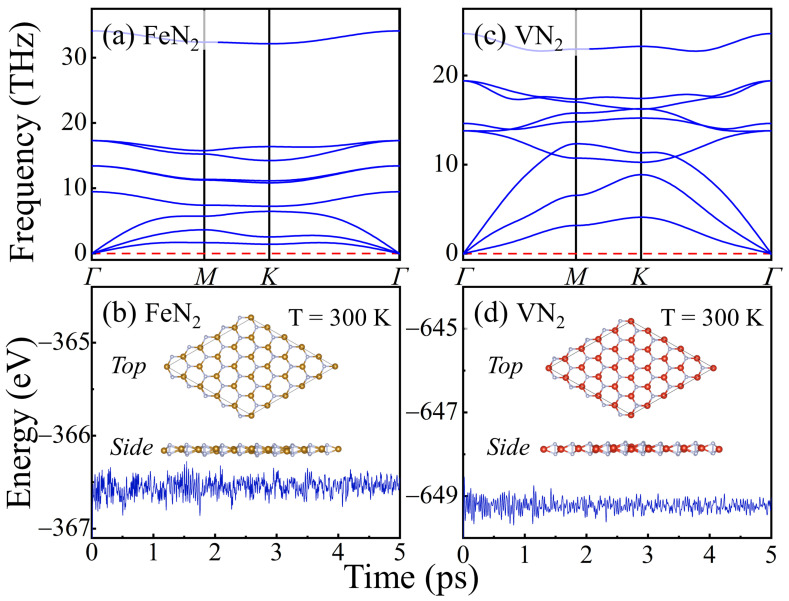
(**a**,**c**), Phonon spectra of FeN_2_ and VN_2_ monolayers in the ferromagnetic ordering phase. (**b**,**d**), Total energy evolution with respect to time at 300 K in the molecular dynamics simulations of FeN_2_ and VN_2_ monolayers. The insets are the top and side views of their final configurations after 5 ps molecular dynamics simulations at the temperatures of 300 K.

The phonon spectra is an important means to judge the structural stability of solid materials. Based on the DFTP method, we perform the calculation of the phonon spectra of FeN_2_ and VN_2_ to inspect its dynamic stability. The highly symmetric points in reciprocal space are Γ (0 0 0), M (0.5 0 0), and K (1/3 1/3 0). If there exists an unstable phonon mode, it is presented as an imaginary frequency. As shown in Figure 2a,c, the absence of imaginary modes in the entire Brillouin zone indicates that the monolayer VN_2_ and FeN_2_ are dynamically stable.

Next, we perform the first-principles molecular dynamic simulations to examine their thermal stability. The temperature is set to 300 K, and the time length is 5 ps. In Figure 2b,d, the total potential energy of the MN_2_ (M = Fe and V) monolayer fluctuates around a certain value, and no distinct drop of energy occurs, which indicates that the monolayer framework is retained at the temperature of 300 K and there is no bond broken. The final configurations of FeN_2_ and VN_2_ monolayer after 5 ps molecular dynamics simulations are shown in the inserted diagrams in Figure 2b,d, reflecting the robustness of the FeN_2_ and VN_2_ structures. Therefore, the 2D MN_2_ (M = Fe and V) monolayers have good dynamic and thermal stability.

### 2.3. Electronic Structure

GGA + *U* is a simple method to deal with the highly localized and strongly correlated electron systems in which the Hubbard *U* parameter has a clear physical significance. To determine the value of Hubbard *U* for a certain material, M. Cococcioni and S. de Gironcoli [28] developed a linear response approach inclosed in the VASP code. By this method, we obtain the Hubbard *U* values of 2.2 eV and 3.42 eV for the FeN_2_ and VN_2_ monolayer.

**Figure 3:**
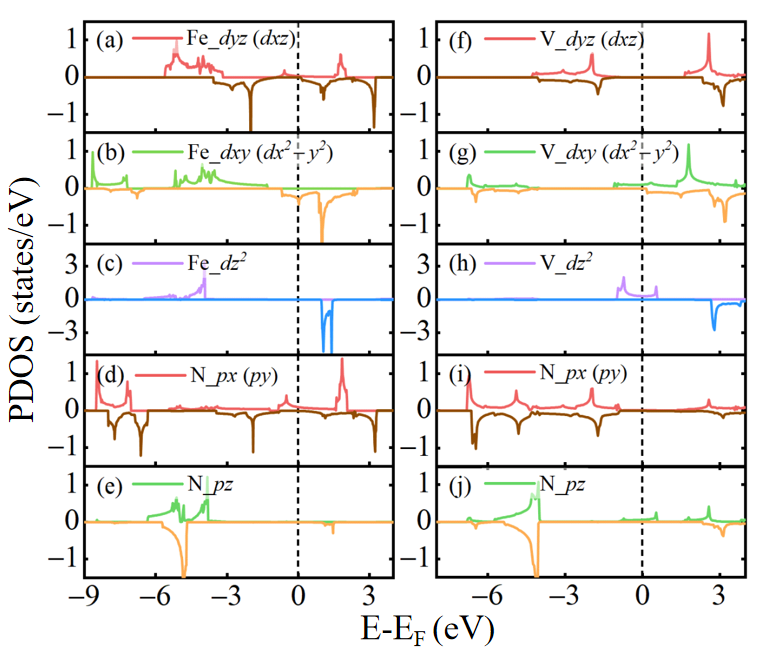
Partial density of states of Fe 3d, V 3d, and N 2p suborbitals in the FeN_2_ and VN_2_ monolayers. (**a**–**c**) $d_{yz}$/$d_{xz}$, $d_{xy}$/$d_{x^{2}-y^{2}}$, and $d_{z^{2}}$ suborbitals of Fe atom in the FeN_2_ monolayer. (**d**,**e**) $p_{x}$/$p_{y}$ and $p_{z}$ suborbitals of N atom in the FeN_2_ monolayer. (**f**–**h**) $d_{yz}$/$d_{xz}$, $d_{xy}$/$d_{x^{2}-y^{2}}$, and $d_{z^{2}}$ of V atom in VN_2_ monolayer. (**i**,**j**) $p_{x}$/$p_{y}$ and $p_{z}$ suborbitals of N atom in the VN_2_ monolayer. The positive and negative values correpond to the spin-up and spin-down channels.

In the hexagonal MN_2_ (M = Fe and V) monolayer, the M atom is in a regular triangular prism and the crystal field of the M atom has the C_3*h*_ symmetry. The five 3d suborbitals are split into three groups, namely $d_{x^{2}-y^{2}}$/$d_{xy}$, $d_{xz}$/$d_{yz}$, and $d_{z^{2}}$, which are labeled as E′, E″, and A′ in terms of symmetry signals in the character table of C_3*h*_. The magnetic moment of Fe or V atom is from the spin moment of unpaired electrons, which is determined by the difference between the number of electrons occupying the spin-up and spin-down states. In Figure 3, the states below Fermi energy are fully occupied and those electronic states above the Fermi energy are empty. For the density of states, it means the number of states per one energy unit (1 eV). By integrating the density of states from the lowest energy to the Fermi energy, we can get the number of occupied electrons. Consequently, for each atomic orbital, the net spin-polarized charge in this orbital is derived and its contribution to the total moment of Fe or V atom is figured out. For the FeN_2_ monolayer, the partial density of states for five 3d suborbitals of the Fe atom is displayed in Figure 3a–c. As shown, these orbitals are strongly spin-polarized. Accordingly, the large moment of 3.2 $\mu_{B}$ is formed around Fe atoms. In the two degenerate E′ orbitals, $d_{x^{2}-y^{2}}$ and $d_{xy}$, all the spin-up states and a small number of spin-down states are occupied, and the electrons in the two orbitals give rise to the magnetic moment of about 1.5 $\mu_{B}$. In the two degenerate E″ suborbitals, $d_{xz}$ and $d_{yz}$, the electrons occupied in the spin-up states are slightly more than the electrons in the spin-down states, and they can give rise to the moment of about 0.5 $\mu_{B}$. As for the $d_{z^{2}}$ suborbital, the spin-up states are fully occupied and the spin-down states are empty, which lead to 1.0 $\mu_{B}$ moment. In addition, there is some spin-polarization in the *s* and *p* states of the Fe atom, which has a small contribution to the total moment. On the other hand, the V atom in the VN_2_ monolayer, the moment is 1.0 $\mu_{B}$. As seen from Figure 3f–h, most of the moment comes from the d$z^{2}$ suborbital and a small part of it is from other *d* suborbitals, namely 0.67 $\mu_{B}$ and 0.33 $\mu_{B}$, respectively.

**Figure 4:**
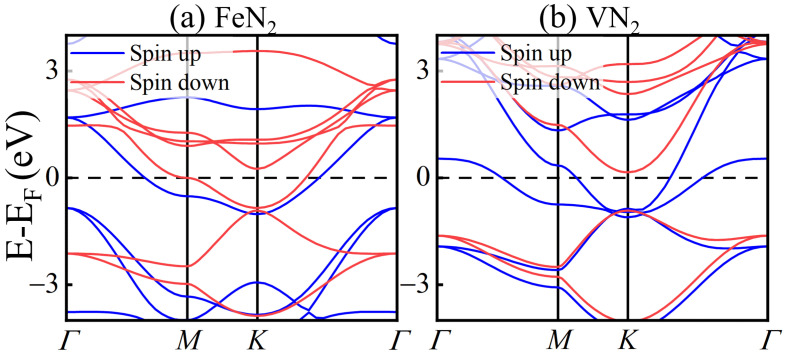
Band structure of FeN_2_ (**a**) and VN_2_ (**b**) monolayers. Spin-up and spin-down bands are displayed as blue and red lines, respectively.

The energy bands of MN_2_ (M = Fe and V) are shown in Figure 4a,b. Their spin-up and spin-down bands have an obvious spin-splitting, and two bands cross the Fermi energy, indicating that the FeN_2_ and VN_2_ are magnetic metals. For the VN_2_ bands, there are only spin-up bands near the Fermi energy, and no spin-down bands appears in the energy range from −0.82 eV to 0.31 eV. So, the 2D VN_2_ acts as a conductor to electrons in the spin-up states, but as a semiconductor to the spin-down electrons. The VN_2_ monolayer exhibits good half-metallicity with a large spin gap of 1.1 eV, which can provide fully spin-polarized currents and large magnetoresistance in some spintronic devices, such as spin-based transistors, diodes, and spin Seebeck devices. For the partial density of states of FeN_2_ monolayer in Figure 3a–e, the spin-down states of Fe *d* suborbitals emerge near the Fermi energy, and the spin-up states of N 2p orbital also exist in the energy range. For the partial density of states of the VN_2_ monolayer in Figure 3f–j, there are only the spin-up states of V *d* suborbitals and N 2p states near the Fermi energy. This results in the half-metallicity appearing in the VN_2_ monolayer instead of the FeN_2_ monolayer.

### 2.4. Ferromagnetism and Curie Temperature

**Figure 5:**
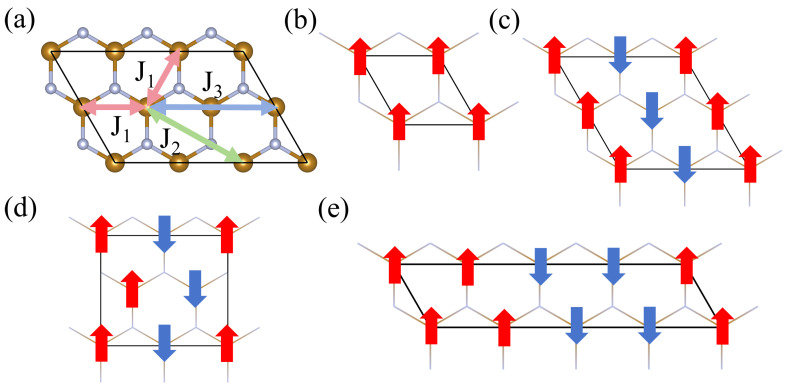
(**a**) The nearest, next-nearest, and next-next-nearest neighbored exchange interaction $J_{1}$, $J_{2}$, and $J_{3}$, marked in the MN_2_ (M = Fe or V) layer with the double headed arrows. (**b**–**e**) FM, AFM-I, AFM-II, and AFM-III orders. The atomic structures are displayed with the wire frame in (**b**–**e**).

**Figure 6:**
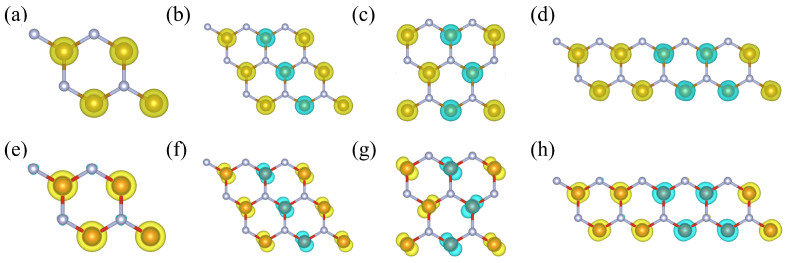
The distribution of spin-charge density in the FM and three AFM orders for FN_2_ and VN_2_ monolayers. FN_2_ monolayer: (**a**) FM, (**b**) AFM-I, (**c**) AFM-II, (**d**) AFM-III. VN_2_ monolayer: (**e**) FM, (**f**) AFM-I, (**g**) AFM-II, (**h**) AFM-III. The yellow and cyan isosurfaces indicate spin-up and spin-down spin density surfaces. The isovalues are 0.057 and 0.018 e/Å_3_ for the FN_2_ monolayer and VN2 monolayer, respectively.

Finally, we demonstrate that the FeN_2_ and VN_2_ monolayers are high-temperature FM materials. In Figure 5, the magnetic couplings between two neighbor moments and three magnetic orders are sketched. For clarity, the atomic structure is displayed with the wire frame in Figure 5b–e. To determine the magnetic ground state of the FeN_2_ and VN_2_ monolayers, the spin-polarized calculations are carried out for three magnetic orders, including the FM order, antiferromagnetic I order (AFM-I), antiferromagnetic II order (AFM-II), and antiferromagnetic III order (AFM-III). The energy per formula cell and the magnetic moment around Fe or V atom are listed in Table 2. In order to present more information on magnetic properties, we illustrate the spin-charge density isosurfaces of FeN_2_ and VN_2_ monolayers in FM and three AFM orders in Figure 6. The isovalues are set to 0.057 and 0.018 e/Å3 for FeN2 and VN2 layers, and these isosurfaces are roughly the same size. The spin density difference, reflected by the two isovalues, is consistent with the difference of magnetic moment, a large moment of 3.3 $\mu_{B}$ around Fe atom and a small moment of 1.0 $\mu_{B}$ around V atom. The yellow and cyan isosurfaces indicate up-spin and down-spin density surfaces. In the AFM-I and AFM-II phases in Figure 6f,g, the distribution of spin-charge density is slightly different from the ones in FM and AFM-III phases in Figure 6e,h, which results in a small change around 1.0 $\mu_{B}$ in the different magnetic orders. The energy differences among FM, AFM-I, AFM-II orders, and AFM-III orders are assumed to only arise from the magnetic interactions. Based on the energy differences, the nearest-neighbor coupling $J_{1}$, next-nearest-neighbor coupling $J_{2}$, and next-next-coupling $J_{3}$ can be derived according to the following expressions,

(2) $$\begin{array}{cc} J_{1} & =\frac{1}{8}\left(E_{FM}-E_{AFM-I}\right)-\frac{1}{4}\left(E_{AFM-II}-E_{AFM-III}\right)\\ J_{2} & =\frac{1}{8}\left(E_{FM}-E_{AFM-I}\right)+\frac{1}{4}\left(E_{AFM-II}-E_{AFM-III}\right)\\ J_{3} & =\frac{1}{16}E_{FM}+\frac{3}{16}E_{AFM-I}-\frac{1}{8}\left(E_{AFM-II}+E_{AFM-III}\right). \end{array}$$

The method for calculating exchange couplings comes from the Appendix in the Ref. [29], which is widely used in the study of ground state magnetism in the magnetic materials [30,31]. The values of $J_{1}$, $J_{2}$, and $J_{3}$ are presented in Table 2. In addition, we compute the single-site magnetic anisotropy energy (MAE) in terms of the definition, $MAE=E_{100}-E_{001}$, and list the values in Table 2. The negative MAE value means the easy magnetic axis is in the ab plane, namely an in-plane magnetic anisotropy.

**Figure 7:**
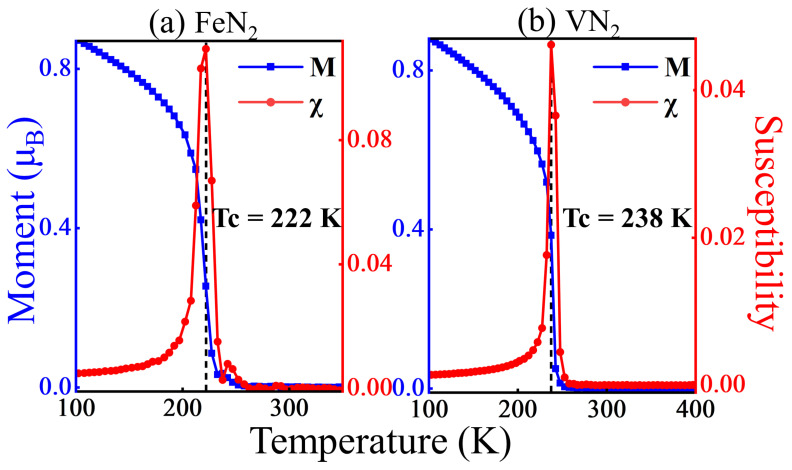
Average magnetic moment *M* and susceptibility χ as functions of temperature. The vertical dashed lines indicate the phase transition temperature.

In previous studies, the 2D Ising model and the Heisenberg model were usually employed to estimate the critical temperature for magnetic phase transition [9,32,33]. For the Ising model in a triangular spin-lattice, the critical temperature is $T_{c}$ = $J_{1}\cdot 4/ln3$ [34]. In terms of the formula, the Curie temperatures of FeN_2_ and VN_2_ monolayers are 830 K and 450 K, respectively, which are overestimated. Ising model is the limit of the Heisenberg model. That is to say, with magnetic anisotropy going to infinity, the Heisenberg model would be reduced to the Ising model. Because of the small magnetic anisotropy in real 2D FM materials, the $T_{c}$ is usually overestimated by the Ising model. Therefore, Heisenberg model is a more precise model and has been successfully used to estimate the $T_{c}$ of CrI_3_ and other synthesized 2D FM materials [35,36]. In this paper, we solve the Heisenberg model with Monte Carlo method to evaluate the critical temperature. The Hamiltonian of the Heisenberg model is defined as

(3) $$\begin{array}{c} H=\sum_{ij\alpha }J_{1}S_{i\alpha }S_{j\alpha }+\sum_{ij^{\prime }\alpha }J_{2}S_{i\alpha }S_{j^{\prime }\alpha }\\ +\sum_{ij^{\prime \prime }\alpha }J_{3}S_{i\alpha }S_{j^{\prime \prime }\alpha }+A\sum_{i}{\left(S_{iz}\right)}^{2} \end{array}$$

in which *j*, $j^{\prime }$, and $j^{\prime \prime }$ represent the nearest, next-nearest, and third-nearest neighboring sites of the i site in the triangle lattice and α is a coordinate component *x*, *y*, or *z*. A is the single-site magnetic anisotropic energy, which is the energy difference when the spin is along the (1 0 0) and (0 0 1) directions. The variation of average magnetic moment and the susceptibility with temperature are presented in Figure 7a,b, where the susceptibility is defined as χ = $\frac{<{\vec{M}}^{2}>-<\vec{M}{>}^{2}}{k_{B}T}$. For the FeN_2_ monolayer and VN_2_ monolayers, the Curie temperatures are 222 K and 238 K, respectively. Among 44 kinds of ferromagnetic 2D materials reported in Ref. [35], the highest Curie temperature predicted by the Heisenberg model is 261 K, which indicates that the FeN_2_ monolayer and the VN_2_ monolayer are high-temperature ferromagnetic 2D materials.

## 3. Computational Methods

The calculations are implemented in the Vienna Ab-initio Simulation Package (VASP) [37,38], and the generalized gradient approximation (GGA) in the form of Perdew-Burke-Ernzerhof (PBE) and the projected augmented wave (PAW) pseudopotential are used [39,40]. The GGA+*U* method [41] is also used to improve the description of the electronic correlation of the Fe and V 3*d* electrons. The criterions of 10^−6^ eV and 0.01 eV/Å are set for the total energy and atomic force convergence. The vacuum layer, plane-wave cutoff energy, and *k*-point mesh are 20 Å, 600 eV, and 39 × 39 × 1, respectively. When the interval between two *k*-points is set to 0.01 × 2π·Å^−1^ in the Brillouin Zone, the number of *k*-points is 39 along *a* or *b* axis. The phonon dispersion is calculated by the density functional perturbation theory (DFPT) method as implemented in the Phonopy code [42]. The first-principles molecular dynamics simulation in the mole volume-temperature (NVT) ensemble of a 5 × 5 × 1 supercell lasts for 5 ps with a time step of 1 fs at 300 K [43].

The temperature of the phase transition is evaluated by solving the Heisenberg model with the Monte Carlo method [44]. Monte Carlo simulations are performed with a 50 × 50 × 1 spin-lattice. For a given temperature, the spin system evolves into a thermal equilibrium state, and the statistical observable such as energy and magnetic moment are directly obtained and other physical quantities can be derived.

## 4. Discussion and Conclusions

It is noted that the space group of VN_2_ and FeN_2_ monolayers is P-6m2, which is a non-centrosymmetric group. In the last decade, topological spin textures such as skyrmions have attracted extensive attention because they have promising applications in novel spintronic devices. Some non-centrosymmetric layered materials have been demonstrated to possess Dzyaloshinskii–Moriya (DM) interaction, which can stabilize the topological spin textures [45,46]. For example, Cr-intercalated hexagonal TaS_2_ with the non-centrosymmetry of P6_3_22 space group and Fe_2.8_GeTe_2_ with the non-centrosymmetry of P6_3_mc group are recently reported. The VN_2_ and FeN_2_ monolayers predicted in this work have similar hexagonal lattices and non-centrosymmetry. Hence, the topological spin textures can be expected to emerge in the MN_2_ (M = V, Fe, and other 3d metals) monolayers.

In summary, by the first-principles calculations, we propose two new 2D transition metal nitrides MN_2_ (M = Fe and V). The stability has been demonstrated by the calculations of phonon spectra, molecular dynamics simulation, and formation energy. The electronic structures showed that VN_2_ monolayers is FM half-metals with a large half-metallic gap. More importantly, the high-temperature ferromagnetism in the FeN_2_ and VN_2_ monolayers are determined through solving the Heisenberg model using the Monte Carlo simulation method. Our work provides a new route to designing two-dimensional materials with intrinsic magnetism.

## Figures and Tables

**Table 1:** The energies of VN_2_ and FeN_2_ with the hexagonal (H), trigonal phase (T), and tetragonal (Tetra) phases.

System	H-Phase (eV)	T-Phase (eV)	Tetra-Phase (eV)
FeN_2_	−22.93576	−21.42701	−22.45834
VN_2_	−25.99425	−25.15685	−24.97182

**Table 2:** The energies in FM, AFM-I, AFM-II, and AFM-III orders, local magnetic moment, exchange coupling $J_{1}$(meV/S^2^), $J_{2}$ (meV/S^2^), and $J_{3}$ (meV/S^2^), MAE(µeV/S^2^) and $T_{c}$ (K) for the monolayer FeN_2_ and VN_2_, respectively.

	E_*FM*_	E_*AFM−I*_	E_*AFM−II*_	E_*AFM−III*_	M	*J*_1_	*J*_2_	*J*_3_	MAE	$T_{c}$
	(meV)	(meV)	(meV)	(meV)	($\mu_{B}$)	(meV/S^2^)	(meV/S^2^)	(meV/S^2^)	(μeV/S^2^)	(K)
FeN_2_	0	86.74	68.45	33.29	3.3	−19.65	−2.07	3.57	−144	222
VN_2_	0	73.71	46.46	40.79	1.0	−10.64	−7.80	2.92	−53	238

## Data Availability

Data are contained within the article.
